# The Challenges of Genome Analysis in the Health Care Setting

**DOI:** 10.3390/genes5030576

**Published:** 2014-07-22

**Authors:** Anneke Lucassen, Richard S. Houlston

**Affiliations:** 1 Clinical Ethics and Law Unit, Wessex Clinical Genetics Service, The Princess Anne Hospital, Southampton, SO16 5YA, UK; 2 Division of Genetics and Epidemiology, The Institute of Cancer Research, Sutton, Surrey SM2 5NG, UK; E-Mail: richard.houlston@icr.ac.uk

**Keywords:** genome sequencing, clinical practice, incidental findings, consent, disclosure

## Abstract

Genome sequencing is now a sufficiently mature and affordable technology for clinical use. Its application promises not only to transform clinicians’ diagnostic and predictive ability, but also to improve preventative therapies, surveillance regimes, and tailor patient treatment to an individual’s genetic make-up. However, as with any technological advance, there are associated fresh challenges. While some of the ethical, legal and social aspects resulting from the generation of data from genome sequencing are generic, several nuances are unique. Since the UK government recently announced plans to sequence the genomes of 100,000 Health Service patients, and similar initiatives are being considered elsewhere, a discussion of these nuances is timely and needs to go hand in hand with formulation of guidelines and public engagement activities around implementation of sequencing in clinical practice.

## 1. Introduction

The speed by which a person’s genome can be analysed has increased phenomenally over recent years, while the attendant costs have plummeted. As a result, genetic testing is shifting from a targeted approach analysing specific genes based on particular symptoms or family histories to sequencing of an entire exome or genome (whole exome sequencing [WES], whole genome sequencing [WGS]). Targeted approaches characteristically have a high yield for penetrant monogenic conditions; whole genome approaches have the potential to unravel a much larger proportion of genetic disease burden. Whole genome analyses, therefore, are likely not only to transform a clinician’s diagnostic and predictive ability, but also to improve preventative therapies, surveillance regimes, and tailor patient treatment to an individual’s genetic make-up.

The improved diagnostic yields of genome sequencing are to be welcomed; however as with any technological advance there are associated fresh challenges. While the ethical, legal and social aspects resulting from the generation of data from genome sequencing are not unique, several nuances merit serious consideration. Since the UK government recently announced plans to sequence the genomes of 100,000 Health Service patients [1], and similar initiatives are being considered elsewhere, a discussion of these nuances is timely and needs to go hand in hand with formulation of guidelines and public engagement activities around the implementation of genome sequencing. Box 1 lists some of the ethical, legal, and social and practical issues that we consider merit consideration.

Box 1 **Some of the overlapping ethical, legal, social and practical issues that need to be addressed as genome analysis enters clinical practice.**

**Complexity**

- Genome analysis can provide many different predictions about diagnoses, or susceptibilities to conditions. However, it will do so with varying degrees of certainty or confidence intervals around the predictions. Such predictions are likely to change substantially over time as evidence about epistatic factors accumulates.
- Providing consent to genomic testing is therefore complex. Should consent be sought to any answer that genome analysis might provide? Or should there be cut-off for levels of certainty? Or should a genome analysis be used solely to answering a current clinical question? Should some results be staged? (e.g., risk of adult onset conditions diagnosed in children?)

**Familial Aspects**

- Although genomic information is on the one hand very personal, on the other, it may be relevant to relatives who have not sought medical advice but may be identified as being at risk from the results in another person. How can health services best record, store and communicate such familial information?

**Re-Contacting/Follow Up Policies**

- Who should be re-contacted and when, in the light of evolving knowledge? Who might be liable if a patient remains unaware of new evidence and therefore interpretation of previous test results?

**Data Management**

- What should be stored: the DNA sample, the DNA sequence, the interpretation of the sequence? Or combinations of these? What is to be stored in medical record systems, and how can these be compliant with relevant data protection—and other—legislation? How can/should these be linked with biobank or research databases, and how can the security issues around identifiable data best be managed?

**Research/Clinical Divide**

- The traditional route of research to clinic evolution is not necessarily applicable in rapidly evolving technologies.

**Public Perceptions of Genetics**

- Currently, this is often thought as a clear cut, or deterministic result than there is evidence for.
- Analytical validity not the same as clinical validity or utility; $1000 genome analysis is a reality soon, yet the cost of interpretation is much greater.

## 2. The Promise of Whole Genome Analysis

Genetic testing has traditionally been restricted to analysing small numbers of genes usually picked on the basis of a high prior probability of being mutated. However, this approach has several limitations. Firstly, many inherited diseases are genetically heterogeneous and sequential mutational analysis of individual genes is slow and expensive. Secondly, while subsets of some common diseases can be caused by mutations in a single gene, traditional methods of selecting whom to test on basis of disease characteristics or family history are crude and have a high false negative rate. Finally, analysis may not ultimately be diagnostic if the disease is a consequence of a hitherto unknown disease-causing gene. Collectively these issues make whole genome approaches at competitive prices an attractive proposition.

Most next-generation sequencing (NGS) technologies are based on the fragmentation of genomic DNA with the oversampling of reads providing the necessary linking information for whole-genome assembly algorithms. For analysis of a gene to be of diagnostic quality using NGS there needs to be sufficient read depth for any mutation to be called with a high degree of confidence. While WGS or WES typically provide good overall coverage for most regions of the genome, for other regions it may be poor; sequencing some regions of the genome is problematic because of repetitive sequence and other features leading to systematic error [2]. Such limitations have, in part, been the motivation for developing targeted sequencing approaches focusing on panels of genes relevant to specific diseases states; for example, cancer gene and nervous system disease panels. Such technical shortcomings are likely to be addressed in the near future so that a “one-stop-shop” test will replace the sequential approaches to genetic diagnoses which were time and labour intensive.

## 3. Analytical Validity *versus* Interpretation of WGS Approaches

Whilst the analytical validity of WGS approaches is high, and improving at a rapid pace, the clinical validity of the output from WGS is much more complex than commonly perceived and the utility has often been evaluated only in very small groups. There is much genome variation that is either: uninterpretable; probably benign; or only pathogenic in certain circumstances, for example, in the presence of as yet unknown epistatic factors. This gap between technological advances and the interpretation of any NGS output, is neatly encapsulated by the phrase “$1000 genome; $1 million interpretation [3], yet, little recognised in the popular discourse around whole genome technologies.

In the clinical setting, certainly in the short term, diagnostic accuracy will therefore continue to depend on additional factors such as clinical history and, therefore, pre-test probability. Attempts to overcome these issues include use of gene panels or analyses of selected portions of the genetic code; an apparently anachronistic step in the evolution of whole genome approaches. However, if WGS approaches are to be used to answer clinical questions, some sort of filtering of sequence output will need to take place. Although targeted approaches are commonplace in health care, this has usually involved a targeting of the investigations. In WGS the targeting will have to be at the analysis stage—the results require targeted analysis—and this raises novel issues about what constitutes a result, what is disclosed to the patient, and what is recorded in a patient’s medical records.

## 4. The Data Interpretation Problem

Much of the misperception about the diagnostic value of genome sequencing results from an oversimplification in which it is assumed there is “a gene” for the condition, when in fact any increase in risk conferred by a mutation may be subtle, or only manifest in the context of specific genetic background or environmental exposure. For many common diseases there are multiple risk factors and while the identification of susceptibility genes has often provided novel insights in disease biology, their clinical utility in an individual may be very low because their predictive power in isolation is very poor.

There is, however, also a risk of over-interpretation even for mutations with seemingly large effects. For affected patients where there is a strong prior probability of the gene mutation being causal because of a positive family history and or specific clinical phenotype, interpretation can be straightforward. However, if mutations are not fully penetrant, there will be carriers in the population who are healthy. Much of our knowledge about the penetrance of mutations to date is based on family data and, hence, suffers from ascertainment bias [4]. Without unbiased knowledge of the effect of mutations, interpretation at the population level will be inherently problematic. Whilst policies to restrict genetic testing to high risk populations were initially driven by budget restraints, and the more widespread availability of testing thought to be an advantage of declining costs, another consequence is that the interpretation of the clinical significance of a mutation is much more difficult if found without the ascertainment bias noted above. That is to say, predicting the effects of a novel *BRCA2* mutation in the context of a strong family history of the mutation segregating with disease in the family, is far easier than when it is discovered in a population screen (see Box 2 for an illustrative example).

Box 2 **Difficulty of clinical interpretation of genomic findings in absence of clear clinical phenotype or family history.**

A two-year old boy was investigated for “absence spells”. He had no loss of consciousness, was investigated in detail for epilepsy and no abnormalities were found. Paediatric cardiologists also found no abnormalities, his baseline ECG was defined as within normal limits and he had no family history (to 3rd degree relatives) of any cardiac problems. The cardiologist had been to a presentation about mainstreaming genetics and realised that long QT (LQT) interval gene carriers can be difficult to detect in childhood. He therefore requested genetic testing of LQT genes “to exclude LQT syndrome”. A LQT1—associated mutation was identified, described on the laboratory report as “highly likely to be pathogenic”. A reveal device was inserted but no abnormalities in his QT interval were recorded during subsequence “absence spells”. Nevertheless, it was thought appropriate to treat him with beta blockers. Cascade testing of his family revealed his three-year-old sister, father, paternal aunt (and her two children, aged four and eight) and paternal grandfather all carried the same mutation. Cardiac investigations of their phenotype, at rest, with exercise, and pharmacological challenge were normal or equivocal. All carriers in the family were prescribed beta blockade and two members of the family were referred for possible implantable cardiac defibrillator insertion.

In Box 2 the assumption that this LQT1 mutation depicts a high future risk of clinical symptoms from LQT syndrome is based on the laboratory description of its likely pathogenicity and the previous finding in families with symptomatic LQT. The intensive therapy is in part because the first presentation of LQT can be sudden cardiac death. However, this family was not ascertained on the basis of any relevant clinical symptoms and clear clinical predictions for the seven asymptomatic carriers are extremely difficult. However, if the mutation was found in a family with a segregating LQT phenotype, preventative therapy would be justifiable on clinical grounds. These cases serve to illustrate that the predictive powers of genetics require more than information about genotype, for the effects of any genotype are dependent on a range of other factors. Importantly, the penetrance of different mutations in the same gene can vary substantially and assigning a likelihood of a mutation being disease-causing will increasingly be based on the synthesis of multiple forms of evidence.

## 5. Determining Clinical Utility of Sequence Variants

The translation of genome sequence into medically actionable information is a key challenge. Without support from segregation in families, assigning pathogenicity can be problematic; notably large duplications, most synonymous and some missense mutations, intronic variants, and most variants in promoter and enhancers are particularly difficult to interpret. Predicting the functional consequences of variants which disrupt protein-coding sequence can also be challenging. A variant might affect a transcription factor binding site, a microRNA target site, affect RNA-splicing or stability or truncate a protein. Finally the issue of linkage disequilibrium (where benign variants lie close to a disease predisposing variant) can complicate interpretation of recurrent risk variants.

Irrespective of whether animal models can adequately mimic human disease such model systems are inherently unsuited to determining the consequences of specific mutations as a routine activity. While yeast and cell line systems can be used to assess the functionality of DNA repair gene mutations the general applicability of such model systems is limited. In view of these factors increasing reliance will be placed on the implementation of *in silico* tools to infer the functional consequences of mutations. Although such algorithms can help to predict the likely pathogenicity of variants, often different tools conclude in opposite directions and without an established relationship between gene dysfunction and disease phenotype, robust risk prediction is problematic.

## 6. The Need to Systematically Catalogue Sequence Variation with Phenotype

Several initiatives are cataloguing and assigning pathogenicity to variants/mutations in various specific genes. Examples of such databases include InSiGHT (International Society for Gastrointestinal Hereditary Tumours Incorporated) [5], LoVd (Leiden open variant database) [6] Decipher [7] and DMuDB [8] (the diagnostic mutation database), and the Locus Reference Genomic Collaboration [9]. These resources provide health care professionals with valuable information for decision making processes. While published reports are valuable sources for such databases their stewardship depends heavily on the submission of individual variants and associated clinic-pathological data by sequencing laboratories using some form of incentivization. Currently these databases are limited to curation of restricted number of genes. Even here translating genomic sequence into medically actionable information can be highly time consuming.

To meet the future needs, comprehensive resources with a far more overarching remit will need to be developed and maintained. This needs to be coupled with adoption of automated machine learning, support vector machines and other technologies to create systematic and efficient mechanisms to assess the impact of variants found by genomic sequencing. All of this will require substantial investment before it becomes a reality and has not been factored into the $1000 genome analysis headlines.

## 7. Diagnostics *versus* Population Screening

Given the significant limitations to our current understanding of the impact of genetic variation, we believe that clinical genome sequencing should for now be focused on particular clinical presentations compatible with a genetic aetiology, rather than engaging in opportunistic population screening. For example, the identification of an *APC* mutation in a person with colonic polyposis is diagnostic and highly predictive for family members. In contrast the identification of variants, such as *LQT1* described in Box 1, in a population screen do not have sufficient certainty to infer as much, resulting in difficult clinical management issues. Such contextual differences may be difficult to grasp if genetics is portrayed as being clear cut, and clinical interventions may therefore be offered without sufficient evidence for their benefit.

Intelligent interrogation of genomic outputs in the clinic should initially therefore be restricted to specific genes or diseases for which there is a high prior likelihood of diagnosis. Any opportunistic screening should in the first instance be limited to known epistatic factors for particular conditions, e.g., low risk genes for breast cancer in the investigation of a family history of breast cancer, and formal evaluation of the benefits should not be leap frogged just because of the rapidly decreasing costs of the technologies involved.

## 8. The Need for Large Scale Genotype-Phenotype Linkages

Before more widespread population genome screening is to be contemplated, large-scale systematic and longitudinal investigation of variants in categorised populations would need to take place and their penetrance robustly determined. Depending on variant prevalence the ongoing international biobank sequencing projects are likely to provide a rich source of such data. Additionally, variants identified through clinical testing or research projects, could together with associated phenotypic information, be submitted to publicly accessible databases cataloguing genomic variation. Many of the current databases are however relatively ad hoc affairs and disease-specific. If the full potential of genomics is to be realised there is a need for the development of big data centres which have an overarching remit. However, the development and establishment of such initiatives brings with it the significant issue of data-storage and allied security requirements. These linkages will have to be undertaken within legislative frameworks relating to data protection within host countries and adapted to any changes to such legislation. For example, proposed changes by the European Commission to the data protection directive may have far reaching consequences for the gathering of such linkages [10].

## 9. The Need for Public-Professional Engagement

In parallel with the acquisition and curation of genetic data there needs to be an ongoing dialogue with health care professionals and the public around understandings and interpretations of genomic data so that expectations of new WGS approaches are realistic and grounded in evidence. In the wake of public anxiety around large scale databases, e.g., care.data in UK [11], this dialogue urgently needs to incorporate the importance of data sharing to realise the clinical utility of whole genome approaches. It also needs to incorporate the issues around shifting the point of targeting, as outlined in Section 3. For example, international recommendations suggest that children should not be offered genetic testing for adult-onset conditions (unless a result would alter their medical management). However, once such a result is available many would opine it should be disclosed, even if they would not have tested for it in the first place [12,13].

## 10. Incidental Findings

Any broad, highly sensitive investigation has the ability to occasionally detect abnormalities that are incidental to the reason for the test. Whole genome approaches are much more likely to detect asymptomatic or silent abnormalities that have nothing to do with the current clinical reason for a test. Such findings have been variably termed “secondary”, “non-pertinent”, “unexpected” or “incidental” belying the fact that the appropriate adjective may vary according to the situation [14]. A genome test can, however, only have an incidental finding (IF) if it is used to answer, for example, a particular clinical question. If the question is “what are the abnormalities in this genome?” then there can be no IFs.

There has been much recent debate about the management of IFs in clinical applications of WG technologies [15,16,17,18,19,20]. The American College of Medical Genetics and Genomics (ACMG) produced guidelines that recommended the active search for particular IFs if using WGS/WES approaches [21]. The heated debate that ensued was largely focused on patient/parental choice regarding such IF searches with their purported “right not to know” being exercised by such guidance. A subsequent amendment now argues for decision about IF search to be made at the time of testing, but still recommends search for additional mutations not indicated by the clinical symptoms. The European Society of Human Genetics (ESHG) responded that WG approaches should be targeted to the clinical question, but there is still widespread debate about the management of IFs in practice and whether real up-front patient choice is feasible or preferable.

## 11. Familial Consequences of IFs

A family history of a particular disease usually means that unaffected relatives have some idea they too might be at risk. In contrast, if something is found incidentally there is unlikely to be awareness of the suspected condition. Furthermore, a new variant may only be found to be clinically significant once it has been studied alongside phenotypes in a family and the absence of a family history is likely to make the need for such cascade screening more difficult to comprehend. Furthermore, professionals may be uncertain what, if any, duties they have to alert relatives about risks that may only be clarified after cascade screening.

## 12. Return of Results from Genomic Testing

As the pace and scale of genetic testing increases, it is inevitable there will be less time to prepare individuals for potential test results. Since the implications of some variants, particularly IFs, may fall outside the expertise of the professional who requested the test, referral to another health care professional may be necessary. This process is likely to add to anxiety of families burdened with unexpected genetic information and means that consent and disclosure practices become dissociated. Training in genomic medicine should be expanded to all medical specialties so that the complexities of genomic information can be adequately communicated but we do not underestimate the size of this task in a rapidly changing environment. We suggest that clinical genetic professionals, although relatively few in number, will need to take on greater liaison activities to facilitate this training.

Opinions about disclosure of IFs vary, ranging from full disclosure to disclosing only those with established clinical significance, and/or which have an intervention can impact on disease. In reality clinically significant, because further investigations of the patient, and their relatives, may be required it can be extremely difficult to withhold details of IFs, even if a conclusion is that they are not to arrive at this conclusion. Even if the pathogenicity of an IF is established, disease onset may not be for many years. Hence robust mechanisms are required to identify, re-contact, and review family members when health care interventions become appropriate. Current health-care systems are, however ill-equipped to deal with the recording of familial information, future risks to health, or the monitoring of multiple family members. We consider that genome results need to be considered as a resource that can be accessed over time [22], rather than as one result that needs to be disclosed as one at the point of testing.

## 13. Consent for Genome Testing

Providing individuals with sufficient information in order to make decisions about investigations or interventions is a key element of good clinical practice. Achieving a balance between providing sufficient information but avoiding overload can be a challenge, especially for tests where multiple different outcomes are possible. Individuals need to understand what genome tests can reveal, but also that some degree of uncertainty is likely. The possible need to investigate relatives to assign pathogenicity of variants found is a difficult issue to incorporate into any consent process. Obtaining adequate consent to disclose an IF for which there is no prior suspicion on the basis of family history or symptoms is likewise problematic, especially if such an IF is unlikely to have clinical consequences for some time. All of this is set against a background of media coverage that generally portrays genetics as clear-cut and highly determinative.

## 14. Is Personalised Medicine a Helpful Term to Promote Genomics?

Although genomic analyses will help to stratify individuals into subpopulations with common characteristics so that particular variants might have greater predictive value, this is not the same as individualisation. A concern about describing genomics as leading to personalised medicine is that it may encourage views of genetic determinism or reductionism. There has been much professional and public discussion regarding which parts of a whole genome sequence should be communicated, with emotive discussions about rights to personal information. On the one hand there is a public perception that some form of medical paternalism might be exhibited where useful information would be withheld, on the other there is acceptance that most of the three billion letter output of a genome sequence has no personal clinical relevance [23]. Some advocate that anyone sequenced should have the right to be appraised of “all of” the test results, even if the clinical relevance is indeterminate. Whilst full disclosure is thought to respect a person’s autonomy it may do the opposite if it delivers outputs that are uninterpretable.

## 15. Conclusions

Whilst the technology of genome sequencing is now a sufficiently mature and affordable technology for it to be implemented clinically, significant challenges around interpretation and implementation remain. We believe that clinical genome analyses should be directed to delivering diagnoses for patients and that integration or linkage with biobanks and other research ventures will be crucial for better clinical translation in the future. We do not underestimate the practical challenges such a statement results in but hope that by delineating some of the complexities and aligning them next to common perceptions of genetics will lead to intelligent international debate about consent and disclosure practices, long-term follow-up arrangements, appropriate communication with relatives and linkages between clinical practice and research.
